# The Development of Hexagonal Boron Nitride Crystal Growth Technologies and Their Applications in Neutron Detection

**DOI:** 10.3390/nano15161256

**Published:** 2025-08-15

**Authors:** Wendong Song, Dan Liu, Fenglong Wang, Lu Zhang

**Affiliations:** 1 Xi’an Yuedengge Technology Co., Ltd., Xi’an 710076, China; songwendong@ydgkj.com.cn; 2 Wuhan Second Ship Design and Research Institute, Wuhan 430064, China; liudan@whhwtech.com; 3 School of Science, Chang’an University, Xi’an 710064, China; 4 School of Advanced Materials and Nanotechnology, Xidian University, Xi’an 710126, China

**Keywords:** hexagonal boron nitride, crystal growth, neutron detection, flux method, wide-bandgap semiconductor, ^10^B enrichment

## Abstract

Hexagonal boron nitride (h-BN), a wide-bandgap semiconductor with excellent thermal stability, high electrical resistivity, and strong neutron absorption capacity, has attracted growing interest in the field of solid-state neutron detection. This review summarizes the progress in h-BN crystal growth technologies, including HPHT, CVD, and flux methods, highlighting their advantages and limitations. Among them, flux growth stands out for its simplicity and scalability in producing high-quality, large-area single crystals. The application potential of h-BN in next-generation neutron detectors is also discussed, along with key challenges such as ^10^B enrichment, crystal quality, and device integration.

## 1. Introduction

With the rapid advancement of modern science and technology, the demand for high-performance semiconductor materials has grown increasingly urgent, particularly in critical areas such as electronics, optoelectronics, energy, and nuclear safety [1,2,3,4]. Hexagonal boron nitride (h-BN), a representative wide-bandgap semiconductor material, has emerged as a prominent research focus in recent years owing to its exceptional thermal stability, outstanding electrical insulation, high thermal conductivity, and excellent mechanical strength. These unique properties have garnered widespread attention from both academia and industry [5,6].

Since its first isolation by Balmain in 1842 via the reaction of boric acid with potassium cyanide [7], BN has captured researchers’ attention for its unique layered structure and outstanding physical properties. In the late 1950s, Wentorf Jr. achieved the next milestone by converting hexagonal BN into its cubic form under extreme conditions [8], laying the groundwork for modern superhard materials. It was not until the early 1960s, however, that reproducible powder- and hot-press synthesis routes enabled broader access to BN ceramics. Over subsequent decades, advances in high-pressure high-temperature (HPHT) synthesis [9], chemical vapor deposition (CVD) [10], metal-flux growth [11], et al., each built upon earlier methods—overcoming limitations in crystal size, purity, and scalability. Although HPHT methods achieved millimeter-scale crystal growth in earlier studies, the associated complexity and cost of the equipment have limited their scalability [12]. In contrast, more recent advancements in atmospheric-pressure metal-flux methods have enabled the growth of centimeter-scale crystals with improved purity and structural integrity, while also offering cost advantages [13]. Meanwhile, CVD has gained increasing industrial appeal due to its relatively simple process and scalability potential [14].

Structurally, h-BN consists of boron (B) and nitrogen (N) atoms arranged in a two-dimensional hexagonal lattice formed through sp^2^ hybridization, with adjacent layers bonded via weak van der Waals forces [15]. This distinctive layered structure imparts h-BN with remarkable physicochemical stability—featuring a melting point of approximately 3000 °C—and allows it to maintain performance under extreme conditions such as high temperature, high pressure, and intense radiation. Moreover, it exhibits superior electrical insulation, corrosion resistance, and lubricating properties, endowing it with immense application potential [16].

A particularly noteworthy characteristic of h-BN lies in its natural enrichment in the ^10^B isotope, which possesses an extraordinarily high thermal neutron capture cross-section (approximately 3840 barns). This makes h-BN a highly promising material for neutron radiation detection [17,18]. Currently, the widely used ^3^He gas-based neutron detectors face critical limitations due to helium-3 (He-3) scarcity and high cost, thereby prompting the urgent need for novel, high-efficiency alternatives [19,20]. Compared with traditional silicon-based or scintillator detectors, h-BN can directly generate large quantities of charge carriers via nuclear reactions, significantly enhancing detection efficiency and energy resolution, while also mitigating self-absorption effects that commonly afflict conventional detectors [21,22,23]. Previous studies have demonstrated that the theoretical neutron detection efficiency of detectors based on ^10^B-enriched h-BN approaches 100%, with experimental efficiencies already exceeding 60%, underscoring h-BN’s great advantages in the development of next-generation high-performance detectors [24,25,26].

**Figure 1:**
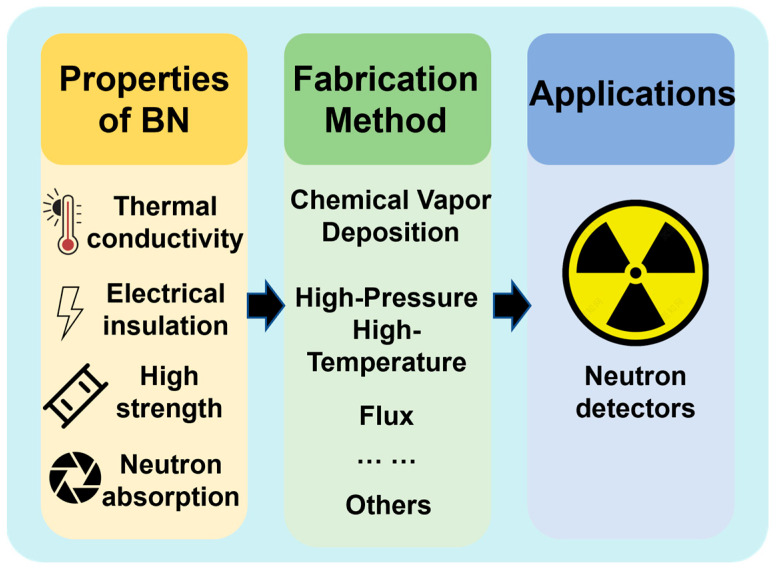
Infographic overview of hexagonal boron nitride (h-BN), summarizing its key material properties, principal fabrication methods, and its application in neutron detectors.

Despite these successes, the practical use of h-BN in neutron detection remains constrained by challenges in growing large-area, defect-free single crystals and by the cost and methods of ^10^B enrichment [27]. In this review, we trace the chronological progression of h-BN growth technologies—from early HPHT experiments through modern flux and vapor-phase methods—placing each advance in its historical context. By doing so, we illuminate how past breakthroughs set the stage for today’s innovations, identify remaining technical bottlenecks, and chart future directions for the industrialization of h-BN–based neutron detectors (Figure 1).

## 2. Introduction of h-BN Materials

### 2.1. Fundamental Properties of BN

**Figure 2:**
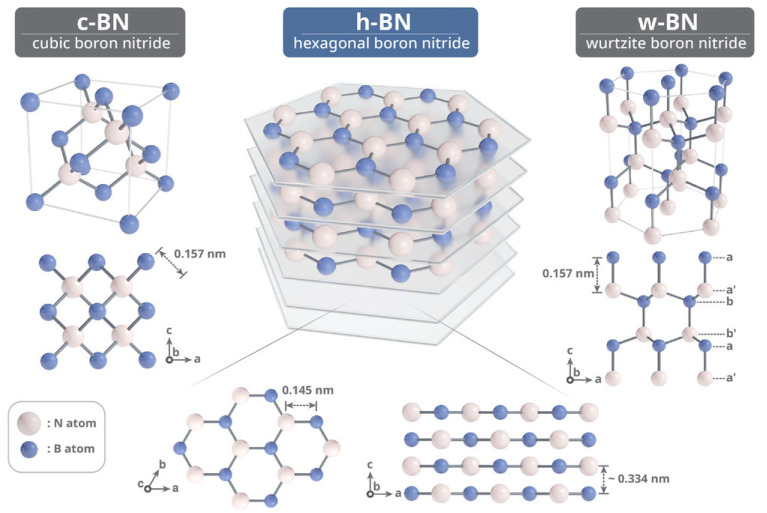
Schematic illustration of the crystal structure of c-BN, h-BN, and w-BN (the image is reproduced from [28], copyright 2022, with permission from John Wiley and Sons).

BN is the simplest compound among group III–V materials and exists in multiple polymorphic forms, including wurtzite (w-BN), sphalerite or cubic (c-BN), hexagonal (h-BN), and rhombohedral (r-BN), as illustrated in Figure 2 [28]. Among these, h-BN was the first polymorph discovered by humans. In 1842, Balmain achieved the first synthesis of h-BN through a reaction involving uric acid and polytrans crystals, marking the earliest known synthesis of BN and establishing h-BN as the foundational polymorph [29]. h-BN is one of the stable BN phases at temperatures over 1000 °C in air, 1400 °C in a vacuum, and up to 2850 °C in an inert gas environment. Over the next century, efforts were made to explore and synthesize other stable BN phases. c-BN is a synthetic material known for its exceptional hardness and structural similarity to diamond. Its first successful synthesis was achieved in 1957 by Wentorf Jr., who transformed boron nitride into its cubic phase under extreme conditions—specifically, high temperatures around 1350 °C and pressures exceeding 6 Gpa [30]. This process, conducted using a belt-type high-pressure apparatus, yielded a material later trademarked as “Borazon”. The transition from the hexagonal (h-BN) to the cubic phase is highly pressure- and temperature-dependent, and the resulting c-BN exhibits mechanical strength closely comparable to that of diamond. The first reported synthesis of w-BN, obtained through the phase transformation of hexagonal BN (h-BN) under extreme conditions of approximately 11.5 GPa and 2000 K, was documented by Bundy and Wentorf, Jr. in 1963, marking an early milestone in the study of high-pressure BN polymorphs [31].

Among all BN polymorphs, h-BN is the thermodynamically most stable under ambient conditions. It crystallizes in a layered hexagonal structure and is often referred to as “white graphite” due to its structural similarity to graphite. The lattice parameters of h-BN are a = 2.504 Å and c = 6.661 Å, with a theoretical density of 2.27 g/cm^3^ and a Mohs hardness of approximately 2 [32]. h-BN melts at around 3000 °C under high pressure. Within each h-BN layer, boron and nitrogen atoms are arranged alternately in a two-dimensional honeycomb network, connected via strong covalent bonds formed by sp^2^ hybridization. These layers stack in an ABAB… sequence and are held together by weak van der Waals forces, which allow them to slide easily over each other—endowing h-BN with excellent solid lubricating properties. This contrasts with c-BN and w-BN, where B–N bonds are sp^3^ hybridized, forming strong three-dimensional tetrahedral networks [33].

The sp^2^ bonding nature within the h-BN layers also contributes to its high thermal stability and high melting point. In addition to its lubricating behavior, h-BN possesses a unique combination of excellent electrical insulation, high thermal conductivity, outstanding chemical inertness, and oxidation resistance (Table 1). It is chemically stable in most environments, showing no significant reaction with water, acids, or bases at room temperature. However, it undergoes slow hydrolysis to form boric acid and ammonia when boiled in water and can react with hot concentrated alkalis or molten bases, as well as chlorine gas at elevated temperatures. h-BN remains thermally stable up to 2000 °C in inert atmospheres [34,35,36].

Due to these unique properties, h-BN has been widely studied and applied in a range of high-performance fields, including electronic packaging, thermal interface materials, radiation shielding, and solid lubrication in harsh environments.

### 2.2. Advantages of h-BN as a Neutron Detection Material

Hexagonal boron nitride (h-BN) has garnered increasing attention in recent years as a promising material for solid-state neutron detection due to its unique physical and chemical properties, including a high thermal neutron capture cross-section, wide bandgap, high resistivity, and environmental robustness [37,38,39]. Compared with conventional neutron detection materials, h-BN offers several notable advantages, making it a strong candidate for next-generation detectors. First and foremost, h-BN naturally contains the isotope ^10^B, which exhibits an exceptionally high thermal neutron capture cross-section (3840 barns) [39]. Upon interaction with thermal neutrons, ^10^B undergoes a nuclear reaction, producing energetic secondary particles such as α-particles and ^7^Li ions. The nuclear reaction between ^10^B and a neutron is shown in Equations (1) and (2). These charged particles deposit their energy within the material, generating a large number of electron-hole pairs, which enables efficient neutron detection [40]. Theoretical studies indicate that an h-BN crystal grown with natural boron achieves near 100% thermal neutron absorption at a thickness of approximately 1 mm. In contrast, h-^10^BN, synthesized with enriched ^10^B, requires only ~200 μm to achieve the same capture efficiency, significantly enhancing detector sensitivity and efficiency.

(1) B510+n01→L37i(1.015 MeV)+α(1.777 MeV)(6%)

(2) B510+n01→L37i*(0.840 MeV)+α*(1.470 MeV)(94%)

Second, h-BN is a wide-bandgap semiconductor with a bandgap of approximately 6.0 eV and an extremely high resistivity (~10^14^ Ω·cm) under undoped conditions. This yields an intrinsically low leakage current, thereby greatly reducing background noise and improving the signal-to-noise ratio in detection systems. The high electrical resistivity also ensures stable operation under strong electric fields, which facilitates the design and fabrication of large-area devices for scalable deployment [36]. Furthermore, h-BN exhibits excellent environmental robustness. The material possesses exceptional thermal conductivity—up to 600 W/m·K in-plane and ~34 W/m·K along the c-axis—as well as a low thermal expansion coefficient [36]. These properties enable h-BN-based detectors to function reliably under extreme conditions, including high temperature, high pressure, and intense radiation environments. This makes h-BN particularly well-suited for applications such as reactor monitoring, aerospace exploration, and downhole logging in the oil and gas industry, where conventional detectors often suffer from thermal and radiation-induced degradation. More importantly, both boron and nitrogen are low atomic number (Z) elements, resulting in weak interactions with γ-rays. As a result, h-BN detectors exhibit strong γ-ray discrimination capability, effectively suppressing γ-induced noise signals [40,41,42]. This selective sensitivity is crucial in scenarios where neutron–γ discrimination is essential, enabling more accurate and reliable detection outputs.

**Figure 3:**
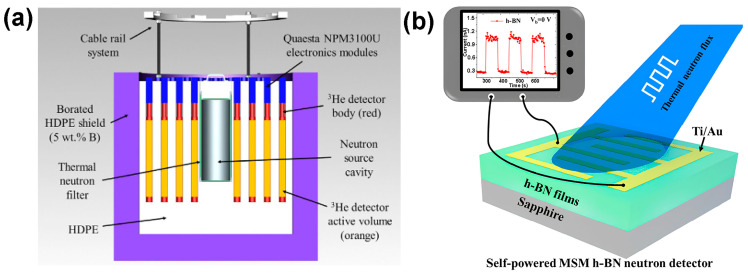
Schematic diagram of thermal neutron detectors. (**a**) ^3^He gas neutron detectors (the image is reproduced from [45], copyright 2023, with permission from Elsevier) and (**b**) h-BN MSM neutron detectors (the image is reproduced from [46], copyright 2021, with permission from American Chemical Society).

Compared to widely used ^3^He gas detectors, h-BN-based detectors offer several practical advantages: they are more compact and lightweight, do not require pressurized gas environments, and operate at low power with minimal maintenance [43,44]. The typical schematic diagram of thermal neutron detectors, including ^3^He gas neutron detectors and h-BN MSM neutron detectors, is shown in Figure 3. These characteristics render h-BN highly advantageous for portable or space-constrained applications, offering substantial benefits in terms of system integration, mobility, and operational cost.

In summary, due to its outstanding physical properties and superior environmental stability, h-BN is emerging as a highly attractive candidate for solid-state neutron detection. As material synthesis techniques continue to evolve and device architectures become more refined, h-BN-based detectors are expected to achieve even greater performance, enabling their widespread application in nuclear physics research, radiation safety monitoring, and resource exploration. The future of h-BN in neutron detection holds immense promise.

## 3. Development of h-BN Crystal Growth Technologies

h-BN, as a wide-bandgap semiconductor with unique physicochemical properties, has demonstrated growing application potential in electronic devices, thermal management, and nuclear radiation detection. However, its ultra-high melting point (~3000 °C) and extremely low vapor pressure pose significant challenges for crystal growth, making it difficult for conventional synthesis techniques to produce high-quality, large-area single crystals. To address these limitations, researchers have developed and refined a variety of advanced synthesis methods, including HPHT techniques, CVD, metal-flux growth, and PVD. These methods have led to remarkable improvements in both the size and quality of h-BN crystals, laying a solid foundation for their application in high-end technologies. This chapter provides a systematic overview of the recent progress in h-BN crystal growth techniques, highlighting the specific features, advantages, and critical challenges associated with each method, with the aim of guiding future research and facilitating the broader application of h-BN materials.

### 3.1. High-Pressure High-Temperature (HPHT) Synthesis

The HPHT method, a classical technique for synthesizing superhard materials, has become a legendary chapter in the field of materials synthesis. Since Wentorf’s pioneering synthesis of cubic boron nitride (c-BN) at General Electric Laboratories in 1961 [47], the HPHT method has undergone over half a century of continuous innovation, repeatedly setting new milestones in the growth of h-BN crystals. This section systematically reviews this remarkable scientific journey from three perspectives: technological evolution, key breakthroughs, and remaining challenges.

The core of Wentorf’s breakthrough lay in the discovery of the “solvent effect” of alkali metal catalysts. Using a magnesium-based catalyst system (Mg–B–N), he successfully achieved the transformation of h-BN to c-BN at 4.5 GPa and 1500 °C [47,48,49]. This not only validated Bundy’s thermodynamic prediction that the pressure term in the Gibbs free energy change (ΔG = VΔP − SΔT) dominates above 4 Gpa [26], but also revealed the crucial role of high pressure in reconstructing sp^2^-hybridized structures. However, due to the technological limitations of high-pressure equipment at the time, the resulting crystal sizes were typically less than 1 mm and exhibited significant inclusion defects. In 1980, Japanese scholar Mishima improved pressure stability by designing a sealed composite structure using pyrophyllite as the pressure-transmitting medium, enabling stable pressure control within ±0.2 GPa and extending the crystal growth duration to over 24 h [26].

**Figure 4:**
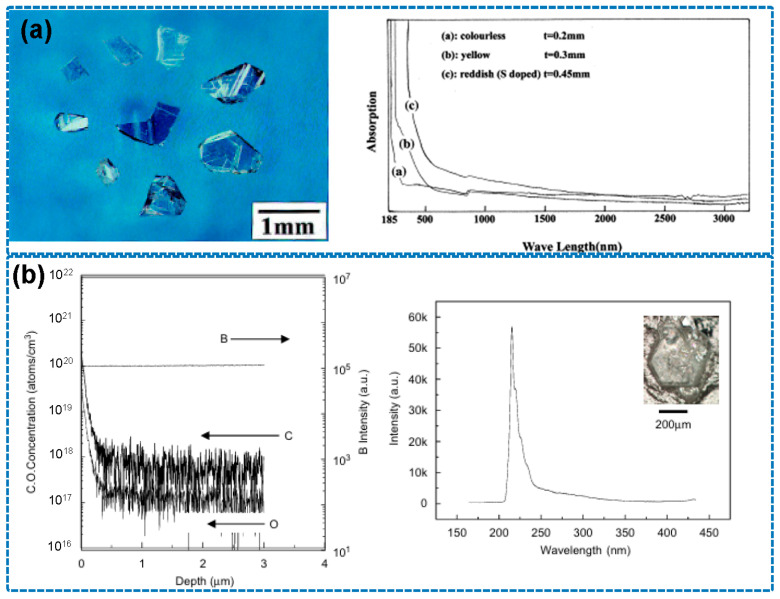
(**a**) Optical image of h-BN single crystals based on Ba–B–N solvent system (the image is reproduced from [50], copyright 2001, with permission from Elsevier), (**b**) SIMS depth profiles and CL spectrum of h-BN crystal (the image is reproduced from [12], copyright 2007, with permission from Elsevier).

Entering the 21st century, a series of studies by the Taniguchi group at the Tokyo Institute of Technology significantly advanced HPHT technology. By developing a Ba–B–N solvent system and conducting synthesis at 1500–1700 °C and 5 GPa, they successfully produced optoelectronic-grade h-BN single crystals with ultraviolet transmittance exceeding 80% [50,51], which is shown in Figure 4a. Through the implementation of gradient purification techniques, oxygen impurity concentrations were reduced below 10 ppm, effectively resolving the long-standing issue of yellowing in h-BN crystals. In 2007, the team further employed an axial temperature gradient method (ΔT = 50 °C/mm), achieving a crystal growth rate of 2 mm/h and breaking the 5 mm size barrier for the first time [12], which can be seen in Figure 4b.

**Figure 5:**
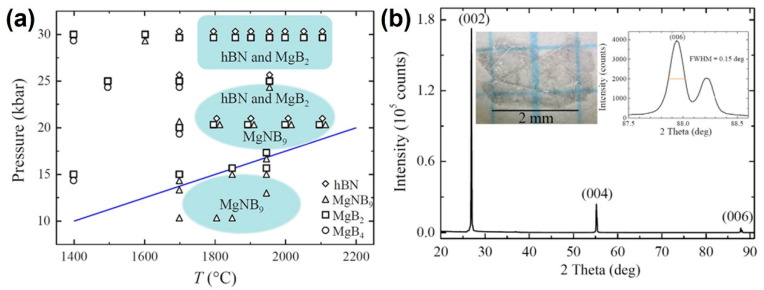
(**a**) Pressure–temperature phase diagram of Mg–B–N system. (**b**) Spectrum of X-ray diffraction of hBN single crystal (left inset) grown at 30 kbar and 2000 °C, the right inset shows a magnified version of the (006) peak (FWHM = 0.151) (the image is reproduced from [52], copyright 2014, with permission from Elsevier).

In 2014, Zhigadlo from ETH Zurich made further breakthroughs by designing a multicomponent Mg–B–N catalyst system. Under 7 GPa and 2000 °C, they successfully grew colorless, transparent h-BN single crystals with diameters up to 2.5 mm and thicknesses up to 10 μm [52]. Raman spectroscopy revealed an FWHM of just 8.0 cm^−1^ at 1367 cm^−1^, a record at the time for crystalline quality. By employing a gradient pressure ramping method (from 5 to 7 GPa in 30 min), they stabilized the growth rate at 0.2 mm/h while maintaining dislocation densities below 10^4^ cm^−2^. Thermal window studies further indicated that the optimal crystal quality was achieved in the 1800–2000 °C range; beyond 2000 °C, reduced nitrogen partial pressure caused deviations in the B:N stoichiometric ratio, leading to the formation of nitrogen vacancies (see Figure 5).

In recent years, research has shifted toward precise process control and defect engineering. The researchers developed an acoustic emission monitoring system capable of real-time tracking of the crystal growth interface by capturing stress wave signals in the 80–120 kHz frequency range. This aligns with the principle of in situ TEM techniques that dynamically resolve stress-induced microstructural evolution [53]. Meanwhile, Kamikawa et al. proposed a two-stage annealing process (1600 °C → 1200 °C with a 5 °C/min cooling gradient), which reduced dislocation densities from 10^8^ to 10^6^ cm^−2^ [54]. Notably, in 2022, Sumiya reported the development of a smart pressure chamber system that utilized shape memory alloys as pressure-compensating elements, successfully reducing residual stress in 10 mm-scale crystals by 70% [55].

Despite substantial progress, HPHT technology still faces three major challenges. First, the lifespan of traditional WC/Co anvils drops sharply above 7 Gpa [56]. Although Anmin Nie et al. developed nanocrystalline diamond anvils that withstand pressures up to 10 GPa, the cost increased nearly tenfold [48]. Second, as crystal sizes exceed 15 mm, thermal stress-induced cracking becomes increasingly severe. Recently proposed multi-zone dynamic pressure compensation techniques have shown promise in mitigating this issue [57]. Third, current catalyst systems show limited efficiency in passivating nitrogen vacancies. The Y_2_O_3_–MgO composite catalyst developed reduced vacancy concentrations by an order of magnitude [58].

With recent advances in precision ultra-high-pressure fabrication—particularly the integration of two-stage amplification systems and smart sensor technologies—HPHT is evolving toward “high-precision, intelligent synthesis” [59]. The digital twin platform recently developed uses machine learning algorithms to dynamically optimize pressure–temperature parameters, boosting the yield of successful crystal growth to 85% [60]. It is foreseeable that this time-honored technology, six decades in the making, will continue to revitalize and drive the frontier of h-BN single-crystal fabrication.

### 3.2. Chemical Vapor Deposition (CVD) Synthesis

CVD is one of the most promising techniques for synthesizing high-quality, large-area h-BN single-crystal thin films. Compared to the conventional HPHT method, CVD offers lower equipment costs, greater process flexibility, and is well-suited for industrial-scale production [61]. In recent years, it has demonstrated considerable application potential in fields such as electronic devices and nuclear radiation detection. The CVD growth of h-BN films typically involves several sequential stages, including substrate pretreatment, decomposition of gaseous precursors, surface diffusion and adsorption of reactive species, and nucleation followed by grain coalescence. The choice of substrate material and its surface treatment significantly influence the quality and orientation of the resulting h-BN films [62,63].

**Figure 6:**
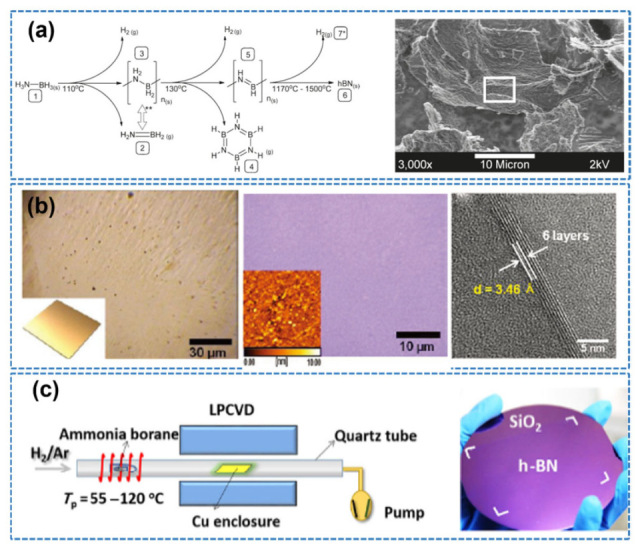
(**a**) Chemical pathways in the pyrolytic decomposition of ammonia borane to boron nitride and FESEM image of BN (the image is reproduced from [64], copyright 2011, with permission from ACS Publications). (**b**) Large-scale synthesis of high-quality h-BN nanosheets in a CVD process by controlling the surface morphologies of the copper (Cu) catalysts (the image is reproduced from [10], copyright 2012, with permission from American Chemical Society). (**c**) Chemical vapor deposition growth of large-scale hexagonal boron nitride with controllable orientation (the image is reproduced from [67], copyright 2015, with permission from Springer Nature).

In 2010, research focused on using ammonia borane as a precursor in atmospheric-pressure chemical vapor deposition (APCVD) to create boron nitride (BN) materials and explore their properties. The solvolysis of ammonia borane can be catalyzed to produce hydrogen [64,65]. Also, the pyrolytic decomposition of ammonia borane can produce boron nitride. Furthermore, metal catalysts, like nickel, can be deposited on boron nitride spheres [66]. Ammonia borane (NH_3_BH_3_) is a promising precursor material in chemical vapor deposition (CVD) for the production of boron nitride (BN) films. The APCVD method was pioneered to be used to synthesize continuous h-BN films with controllable thickness (5–50 nm) on nickel foil using ammonia borane (NH_3_–BH_3_) as the precursor (Figure 6a). The resulting films exhibited near-stoichiometric B/N ratios and an optical bandgap of approximately 5.92 eV [64]. However, the crystallinity was severely constrained by the polycrystalline nature of nickel foil, where surface roughness exceeding 10 nm RMS and random grain orientations (e.g., mixed (110)/(100) domains) disrupted epitaxial alignment. This aligns with the established limitation that polycrystalline nickel substrates induce multidomain h-BN growth with high-density grain boundaries.

To overcome the limitations of Ni substrates, Kim et al. (2012) pioneered the use of low-pressure CVD (LPCVD) on electrochemically polished copper foil to enhance surface flatness (RMS < 5 nm) and grain size (>100 μm) [10], enabling monolayer h-BN films with micrometer-scale grain fusion (Figure 6b). Their work highlighted the dominant role of surface diffusion kinetics over catalytic activity in h-BN film growth, significantly advancing the understanding of CVD mechanisms. Subsequently, Song et al. (2015) introduced a confined-space growth strategy using Cu foil, which effectively reduced the precursor supply rate and nucleation density [67]. This approach enabled triangular single-crystal domains with edge lengths over 70 μm (Figure 6c). The study confirmed the strong epitaxial alignment dependence on Cu crystallographic planes, with Cu (111) planes favoring > 99% unidirectional h-BN growth due to minimal lattice mismatch (1.7%)—a finding consistent with density functional theory (DFT) predictions.

**Figure 7:**
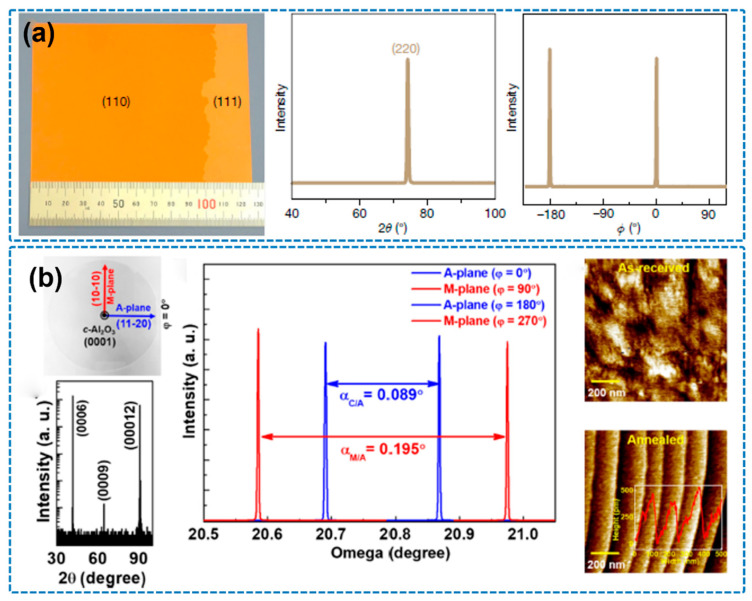
(**a**) Epitaxial growth of a 100-square-centimeter single-crystal hexagonal boron nitride monolayer on copper (the image is reproduced from [68], copyright 2019, with permission from Springer Nature). (**b**) Unidirectional domain growth of hexagonal boron nitride thin films on Cu foil (the image is reproduced from [69], copyright 2023, with permission from Elsevier).

In recent years, significant progress has been made in fabricating large-area h-BN single-crystal films via CVD by optimizing copper substrate treatments. For instance, Wang et al. (2019) utilized industrial-grade copper foil subjected to refined annealing and employed a uniquely tilted Cu (110) crystal plane (Figure 7a), achieving the growth of monolayer h-BN films with areas exceeding 100 cm^2^ and over 99% orientation alignment [68]. This effectively eliminated the anti-parallel domain issue commonly observed on Cu (111) substrates. In 2023, Biswas et al. further expanded the scope of CVD by combining it with pulsed laser deposition (PLD), enabling the direct growth of highly oriented h-BN single-crystal films on insulating sapphire substrates without the need for catalytic metal layers (Figure 7b). By precisely controlling the terrace structure (RMS < 0.2 nm) and interfacial energy of the substrate, unidirectional domain alignment was achieved, yielding excellent crystal quality and promising device-level properties. This work provides a new pathway for fabricating high-performance two-dimensional electronic devices [69].

Despite these significant advances, several technical challenges continue to hinder the industrial deployment of CVD-grown h-BN, including the complexity of preparing large-area single-crystal Cu substrates, difficulties in controlling grain boundaries and associated defects, and damage or reliability issues during the film transfer process [70,71,72]. Future development of CVD technology should thus focus on the following key directions: (1) further refinement of Cu substrate preparation and treatment, particularly in controlling crystallographic orientation and surface smoothness; (2) in-depth investigation of grain coalescence and grain boundary defect mechanisms, enabling high-quality film synthesis through optimized growth parameters; and (3) the development of reliable, low-damage film transfer techniques to ensure the scalability of h-BN thin films in electronic device applications. With continued progress in these areas, the CVD method is expected to play an increasingly vital role in the large-scale production of high-quality h-BN films, supporting broad applications in nuclear radiation detection and next-generation electronic devices.

### 3.3. Flux Growth Method Synthesis

The flux growth method has emerged as an effective approach for synthesizing high-quality h-BN single crystals under ambient pressure and relatively moderate temperatures. Unlike the high-pressure high-temperature (HPHT) technique, which requires extreme pressure-temperature conditions and costly, complex equipment, the flux method utilizes molten metals—such as Ni, Cr, Fe, and Cu—as solvents to dissolve boron- and nitrogen-containing precursors. Upon slow cooling, h-BN crystals precipitate from the supersaturated solution. Owing to its simplicity, controllability, and cost-effectiveness, this technique has become a mainstream method for the research and synthesis of high-quality h-BN crystals.

**Figure 8:**
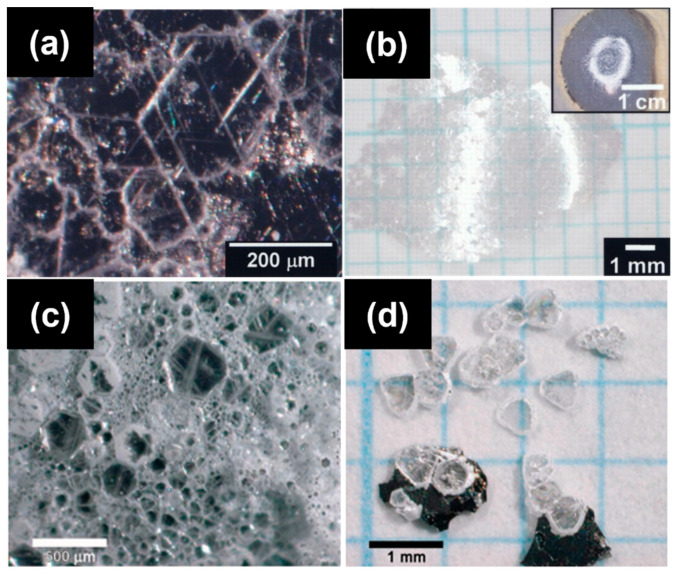
(**a**,**b**) Optical micrographs of recrystallized hBN obtained with a Ni-Mo solvent (the image is reproduced from [73], copyright 2007, with permission from The American Association for the Advancement of Science). (**c**,**d**) Optical micrograph of hBN single crystals obtained with a Ni-Cr solvent (the image is reproduced from [16], copyright 2007, with permission from American Chemical Society).

In 2007, Kubota et al. first reported the growth of h-BN single crystals at atmospheric pressure using a Ni-based flux. By employing a Ni–Mo alloy and cooling the system from 1350–1500 °C to 1200 °C at a slow rate (4 °C/h), they successfully obtained crystals with lateral sizes around 300 μm and thicknesses of several microns [73]. They found that pure Ni had limited nitrogen solubility, while the introduction of Mo significantly enhanced nitrogen incorporation, resulting in larger and thicker crystals (Figure 8a,b). Further improvement was achieved by alloying Ni with Cr, which has even higher nitrogen solubility than Mo, leading to crystal sizes up to 500 μm and thicknesses of 60 μm. Kubota et al. further explored the Ni–Cr system and successfully obtained crystals with diameters up to 500 μm and thicknesses of approximately 60 μm [16]. They attributed this improvement to the significantly higher nitrogen solubility in the Ni–Cr system compared to the Ni–Mo system (Figure 8c,d).

**Figure 9:**
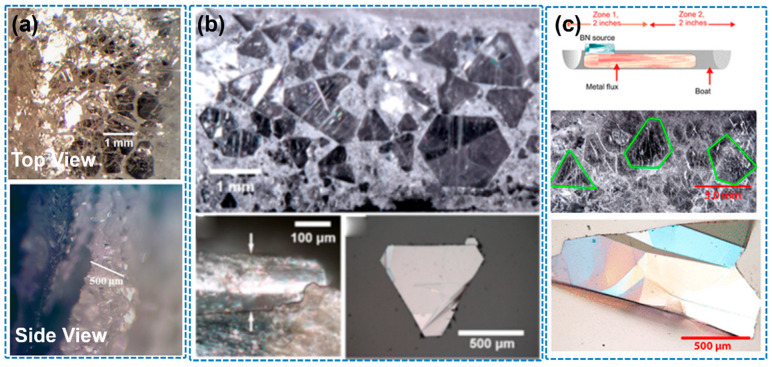
(**a**) Optimization of Ni–Cr flux growth for hexagonal boron nitride single crystals (the image is reproduced from [30], copyright 2014, with permission from Elsevier). (**b**) Optical micrograph of hBN crystals on Fe−Cr ingot top (the image is reproduced from [74], copyright 2017, with permission from American Chemical Society). (**c**) hBN bulk single-crystal growth from metal flux with a temperature gradient (Fe−Cr ingot) (the image is reproduced from [75], copyright 2020, with permission from American Chemical Society).

Building on this foundational work, Hoffman et al. (2014) optimized the Ni–Cr flux system by increasing the peak dwell temperature to 1700 °C and reducing the cooling rate to 2 °C/h, yielding h-BN crystals with lateral dimensions of up to 5 mm (Figure 9a) [30]. These crystals exhibited Raman peaks with full width at half maximum (FWHM) values as low as 8 cm^−1^, indicating low defect and impurity levels. However, the use of graphite heating elements posed a risk of carbon contamination. To address cost concerns, Liu et al. (2017) proposed a more economical Fe–Cr flux system, which yielded crystals up to 2 mm in size with a Raman FWHM of 7.8 cm^−1^—comparable in quality to those grown using the Ni–Cr system (Figure 9b) [74]. In 2020, Li et al. further combined slow cooling with a temperature gradient approach, achieving high-quality h-BN single crystals of 3–4 mm with a record Raman FWHM of 7.6 cm^−1^ for natural isotope boron sources (Figure 9c) [75].

**Figure 10:**
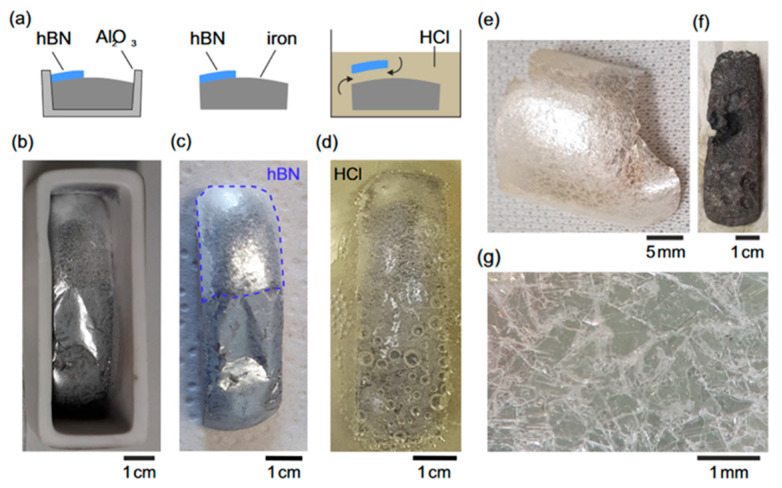
(**a**) Schematic diagram of the hydrochloric acid-based separation process; (**b**–**d**) flowchart of the step-by-step separation procedure using hydrochloric acid; (**e**) optical image of the extracted h-BN crystal alongside the residual etched iron; (**f**,**g**) optical microscopy images of a selected region on the h-BN crystal (the images are reproduced from [77], copyright 2023, with permission from IOP Publishing).

Notably, in 2021, Li et al. reported the first growth of highly ordered polycrystalline h-BN sheets with an area of 4 cm^2^ using a pure Fe flux. Although nitrogen solubility in Fe is relatively low, precise cooling control (1550–1450 °C at 4 °C/h) enabled the successful growth of large, high-quality crystals, thus expanding the applicability of flux-based growth [76]. In 2023, Ouaj et al. further optimized the pure Fe flux system, achieving curved h-BN flakes with areas of 3 cm × 3 cm. As shown in Figure 10, these crystals could be separated from the flux using a simple HCl etching step, demonstrating significant potential for integration into electronic and optoelectronic devices [77].

In the realm of isotopically enriched h-BN crystals, Hoffman et al. (2014) initially attempted to synthesize h-^10^BN and h-^11^BN single crystals using the Ni–Cr flux system, but the resulting sizes were limited to 20–30 μm with broader Raman peaks (FWHM = 14.1 and 9.4 cm^−1^) [78]. Subsequently, Liu et al. (2018) introduced hydrogen protection and reduced the cooling rate to 0.5 °C/h, successfully increasing the crystal size to 1 mm and improving the FWHM to 3.1–3.3 cm^−1^ [25]. In 2020, Li et al. employed an Fe–Cr flux to grow h-BN crystals with lateral sizes up to 5 mm and Raman FWHM as low as 2.7–3.1 cm^−1^ [79]. Other research teams also contributed to this field; for example, Zhang et al. (2021) utilized a Cu–Cr flux with a cooling rate of 2.5–10 °C/h in the range of 1700–1500 °C to synthesize high-quality crystals up to 6 mm in size, with a Raman FWHM of 9.3 cm^−1^ [80]. Y. Li further investigated the influence of boron concentration in pure Fe flux and identified an optimal composition of 16.2 at%, which yielded crystals as large as 400 μm [81].

Overall, the flux growth method demonstrates great potential for the scalable and economical production of large, high-quality h-BN single crystals. Nonetheless, achieving true industrial-scale production will require further refinement of flux compositions, precise control over cooling profiles, reduction in impurities, and continued improvements in crystal size and structural perfection.

### 3.4. Other Synthesis Techniques

In addition to the aforementioned approaches for synthesizing h-BN, several other important approaches have been developed in recent years, including mechanical exfoliation, molecular beam epitaxy (MBE), powder synthesis methods, and magnetron sputtering. Mechanical exfoliation, originally popularized for the isolation of monolayer graphene, has also been applied to the preparation of h-BN crystals. The technique relies on applying external mechanical force to overcome the weak van der Waals interactions between layers in bulk h-BN, thereby peeling off monolayer or few-layer two-dimensional flakes [82,83,84]. Although this method is simple, cost-effective, and does not require sophisticated equipment, it suffers from extremely low yield, poor reproducibility, and difficulty in producing continuous large-area films. As such, it is primarily used in laboratory-scale studies, particularly in fundamental research on two-dimensional materials.

Molecular beam epitaxy (MBE) is a highly precise thin-film deposition technique conducted under ultra-high vacuum. In this process, atomic or molecular beams are directed onto a heated substrate, allowing for the growth of high-purity and high-crystallinity films [85,86]. Recent studies have shown that h-BN films grown on highly oriented pyrolytic graphite (HOPG) substrates via MBE exhibit good crystalline quality [87,88]. However, the high cost of equipment, low growth rate, and complex maintenance limit the scalability of this technique, making it more suitable for fundamental research and high-precision device fabrication.

Powder-based methods mainly include carbothermal reduction and the urea–borax route. In the carbothermal reduction method, B_2_O_3_ and carbon powder are mixed and reacted at high temperatures under a nitrogen atmosphere to form h-BN powders [89,90,91]. Despite its simplicity, the resulting product often contains various impurities, and the purification process is labor-intensive, which has led to a decline in its use. In contrast, the urea–borax method has become the mainstream industrial technique for producing h-BN powders due to its simplicity and high yield [92,93,94]. In this process, urea and borax are mixed and reacted under an ammonia atmosphere at elevated temperatures, producing h-BN powders with purities exceeding 97%, which are widely used in industrial applications such as lubricants and thermally conductive fillers, which is summarized in Table 2.

Magnetron sputtering, a form of physical vapor deposition (PVD), has also been utilized to deposit h-BN thin films. Typically performed at relatively low temperatures, this method involves sputtering a boron target in a nitrogen or nitrogen-argon atmosphere [95,96]. Due to the low substrate temperature, the initially deposited BN films are often amorphous and require subsequent high-temperature annealing to improve crystallinity. However, even after post-treatment, the crystal quality of h-BN films produced by magnetron sputtering remains limited. As a result, their use is primarily confined to functional or protective coatings rather than applications requiring electronic-grade material quality.

One of the primary advantages of the CVD method lies in its ability to precisely control film thickness and surface quality. However, in practical applications, CVD often encounters challenges such as inconsistent crystal quality, high defect density, and difficulties in obtaining large-area single crystals. HPHT techniques can yield high-purity, low-defect single crystals, but they require extreme operating conditions and expensive, large-scale equipment and are prone to impurity incorporation, limiting their widespread adoption. Mechanical exfoliation is suitable for isolating monolayer h-BN from bulk layered crystals, but it is not capable of producing large-area single crystals and offers limited control over crystal quality. MBE enables the growth of high-quality h-BN films at elevated temperatures, yet its high equipment cost and low yield significantly restrict practical application. Traditional powder-based synthesis methods have largely been phased out or confined to niche applications due to their complex product compositions and difficulties in post-processing. In contrast, the flux growth method has emerged as a promising approach for producing high-quality h-BN single crystals under more accessible and cost-effective conditions. This technique has demonstrated the capability to yield large-area, high-crystallinity h-BN single crystals, meeting the increasing demands of advanced applications. Notably, flux growth can be conducted under ambient pressure, eliminating the need for high-pressure apparatus and reducing both operational complexity and equipment costs. Moreover, by selecting appropriate metal solvents and carefully controlling the cooling rate, the flux method enables precise regulation of crystal size and quality. Additionally, it offers the potential for scaling up crystal size, making it particularly well-suited for the synthesis of large, high-quality h-BN single crystals. In summary, the flux method holds distinct advantages and has proven to be a highly effective strategy in the field of h-BN crystal growth.

In summary, while these alternative synthesis techniques each present unique advantages and limitations, they collectively contribute to the diversified development of h-BN in both fundamental research and industrial applications. Moving forward, with continued technological advancements, these methods are expected to play important roles in specific application scenarios. Nevertheless, the development of scalable, low-cost processes capable of producing large-area, high-quality h-BN crystals remains a key research priority in this field.

## 4. Applications of h-BN in Neutron Detection

**Figure 11:**
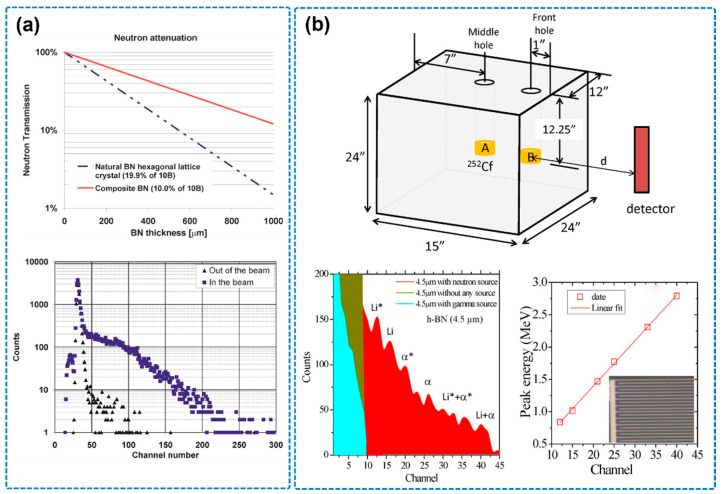
(**a**) Attenuation of thermal neutrons in BN hexagonal crystal and pulse height spectrum squares obtained with a device biased to 450 V and illuminated by thermal neutrons (the image is reproduced from [97], copyright 2007, with permission from AIP Publishing). (**b**) Schematic diagram of thermal neutron source consisting of a ^252^Cf source moderated by a high-density polyethylene (HDPE) moderator and nuclear reaction pulse height spectrum, noting that Li* (0.840 MeV), α* (1.470 MeV), Li (1.015 MeV), and α (1.777 MeV) (the image is reproduced from [15], copyright 2016, with permission from AIP Publishing).

h-BN has attracted increasing attention in recent years due to its excellent neutron detection performance. Significant progress has been made in various aspects of h-BN-based neutron detectors, including structural design, material synthesis, and performance optimization. In 2007, J. Uher et al. first incorporated natural boron particles (with a ^10^B content of approximately 10%) into a polycrystalline semiconductor matrix to develop a composite BN neutron detector (Figure 11a) [97]. Monte Carlo simulations revealed a remarkable neutron capture efficiency of up to 88.6%, far exceeding that of conventional ^3^He gas detectors (2.6%–63.7%). This pioneering work demonstrated the great potential of BN-based materials for neutron imaging and large-area sensing applications. In 2008, the LiCausi research group proposed a novel honeycomb-structured continuous p–n junction neutron detector [98]. By embedding enriched ^10^B material into a silicon substrate, they achieved a detection efficiency of up to 48%, highlighting the critical role of structural engineering in improving device performance.

Since 2016, advancements in thin-film growth technologies—particularly metal-organic chemical vapor deposition (MOCVD) and chemical vapor deposition (CVD)—have enabled breakthroughs in the performance of h-BN neutron detectors. The group led by T. C. Doan employed MOCVD to grow enriched ^10^B h-BN films on sapphire substrates (Figure 11b) [15]. With a thickness of only 2.7 μm, they successfully characterized the carrier mobility–lifetime (μτ) product and achieved a thermal neutron detection efficiency of 4%, with a high charge collection efficiency of 83%. This work provided the first experimental verification of the applicability of epitaxial h-BN layers in neutron detection. In the same year, K. Ahmed’s team utilized CVD to fabricate high-quality h-BN films on both planar (111) Si substrates and vertical trench structures, constructing metal–semiconductor–metal (MSM) detectors [99]. They innovatively developed a completely dry shadow masking process to overcome film delamination issues associated with conventional wet processing, enabling the growth of h-BN films up to 15 μm thick. The resulting detectors achieved neutron detection efficiencies close to the theoretical maximum (~92%), demonstrating that improved crystal quality directly correlates with enhanced device performance.

**Figure 12:**
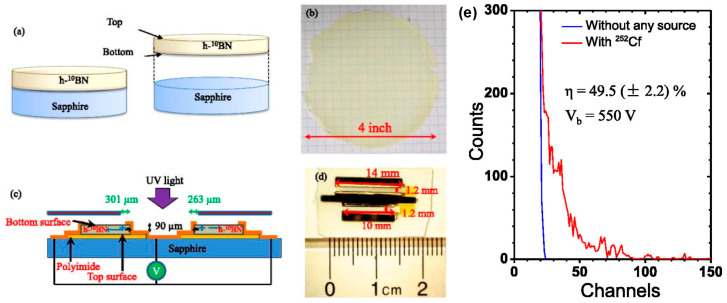
(**a**) Schematic illustration of the separation process used to obtain freestanding h-^10^BN epilayers. (**b**) Optical image of a freestanding h-^10^BN wafer with a diameter of 4 inches. (**c**) Schematic representation of a lateral neutron detector structure, showing the device mounted on an insulating sapphire substrate with a defined active area near one of the metal contacts. (**d**) Optical image of the fabricated neutron detector based on the freestanding h-^10^BN layer. (**e**) Pulse height spectrum of the lateral h-BN detector in response to nuclear reactions, illustrating its detection capability (the image is reproduced from [100], copyright 2019, with permission from AIP Publishing).

In 2018, A. Maity et al. further developed photoconductive neutron detectors using enriched ^10^B h-BN films, achieving detection efficiencies as high as 53% [40]. They systematically investigated the effects of different electrode materials and device architectures, finding that Ni/Au bilayer ohmic contacts combined with negative bias yielded the highest performance. The group also fabricated thick (90 μm) h-^10^BN films and designed lateral detection structures, achieving detection efficiencies of ~50% [100] (see Figure 12). These developments laid a solid foundation for the fabrication of large-area, high-sensitivity h-BN-based neutron detectors. Furthermore, detectors with a detection area of 3 mm × 3 mm were fabricated from 50 μm thick freestanding and flexible ^10^B-enriched h-BN films, maintaining a detection efficiency comparable to previous achievements [101]. By 2024, the thermal neutron detectors based on 100 μm thick ^10^B-enriched h-BN quasi-bulk reached a record efficiency of 60% [102]. Furthermore, a BN detector with a large detection area of 2.1 cm^2^ demonstrated the feasibility of detecting fast neutrons, broadening the application scope of these devices [103].

h-BN’s performance as a neutron detector can be benchmarked against traditional technologies, such as ^3^He gas detectors, scintillators (e.g., NaI (Tl), plastic scintillators), and boron-coated silicon detectors. Table 3 summarizes key metrics. h-BN neutron detectors are characterized by detection efficiency, energy resolution, and timing resolution. Efficiencies range from 40% to 96% for thermal neutrons, surpassing ^3^He (80–90%) in compact configurations [40]. Energy resolution, however, is suboptimal (5–10%) [15,97,100], compared to 1–2% for silicon or 5–10% for scintillators, due to defects and non-uniform ^10^B enrichment [40]. Timing resolution, estimated at 10–100 ns, is adequate for low-event-rate applications but slower than silicon (<10 ns) due to higher energy per pair (20 eV) and defect-related trapping [97]. Table 3 compares h-BN with traditional detectors: h-BN’s compact design and radiation hardness (1000 °C) make it ideal for harsh environments, unlike scintillators, which degrade under radiation. ^3^He’s large volume suits portal monitors, but its scarcity favors h-BN. Silicon’s speed is superior, but its low intrinsic neutron sensitivity requires boron coatings [20].

Researchers are actively investigating advanced fabrication methods like mechanical exfoliation and chemical separation to enhance detector performance by producing large-area hexagonal boron nitride (h-BN) single crystals; however, obstacles such as complex synthesis procedures, maintaining purity, minimizing crystal defects, and achieving scalable production impede widespread industrial use [26,104]. High-quality h-BN single crystals are desirable because of their exceptional properties, including high thermal conductivity, chemical inertness, and mechanical strength, making them suitable for various applications, including dielectrics in electronic devices, deep-ultraviolet emitters, and neutron detectors [102,105,106]. Overcoming the technical challenges in producing these crystals at an industrial scale is critical for realizing their full potential.

Overall, while h-BN neutron detectors have demonstrated outstanding performance in terms of response time, sensitivity, and detection efficiency, several key challenges remain. These include scalable fabrication of large-area single crystals, cost-effective enrichment of ^10^B, and further optimization of device structures. Future research should focus on overcoming these technical bottlenecks to improve material quality and manufacturing efficiency, ultimately enabling widespread application of h-BN neutron detectors in nuclear safety monitoring, radiation protection, and advanced nuclear technologies.

## 5. ^10^B Enrichment Methods and Considerations

Hexagonal boron nitride (h-BN) is a versatile material prized for its high thermal conductivity, low thermal expansion coefficient, and environmental robustness, making it suitable for applications in thermal management and nuclear shielding. The inclusion of isotopically enriched ^10^B in h-BN enhances its performance in specific contexts, such as neutron absorption, due to ^10^B’s high neutron capture cross-section (~3840 barns) compared to natural boron (~760 barns) [17]. This section discusses the methods for ^10^B enrichment, their impact on h-BN properties, and associated cost considerations.

### 5.1. Enrichment Methods

The enrichment of ^10^B typically involves separating the naturally occurring isotopes of boron (^10^B, ^11^B), which exist in a natural abundance of approximately 19.9% and 80.1%, respectively. Two primary methods are employed for ^10^B enrichment:

Chemical Exchange Distillation: This method utilizes the differential volatility of boron compounds, such as boron trifluoride (BF_3_), in a chemical exchange process. The lighter ^10^B isotope forms slightly stronger bonds, leading to a small separation factor. Industrial-scale distillation columns are used to achieve high enrichment levels, often exceeding 90% ^10^B [36,107]. This method is widely used due to its scalability, though it requires significant energy input and complex infrastructure.

Laser-Based Isotope Separation: Techniques such as laser-induced selective excitation exploit isotopic differences in vibrational energy levels of boron-containing molecules. For example, infrared laser irradiation of BF_3_ can preferentially excite ^10^B-containing molecules, enabling their separation [108]. This method offers high precision but is less common industrially due to higher costs and lower throughput compared to chemical exchange.

These methods produce ^10^B-enriched boron precursors, which are then used in h-BN synthesis via processes like chemical vapor deposition (CVD), as discussed in earlier sections.

### 5.2. Cost Considerations

The cost of ^10^B enrichment is a critical factor in its adoption for h-BN applications. Chemical exchange distillation, while scalable, requires significant capital investment in distillation columns and energy for operation, contributing to higher costs for high-purity ^10^B. Laser-based methods, though more precise, are cost-prohibitive due to specialized equipment and lower throughput [107]. According to industry estimates, enriched ^10^B can cost several hundred dollars per gram, depending on purity and scale, significantly higher than natural boron precursors. These costs impact the economic feasibility of ^10^B-enriched h-BN, particularly for large-scale applications like thermal substrates in electronics, where natural hBN may suffice. However, for niche applications like neutron shielding, the enhanced performance justifies the cost.

In summary, ^10^B enrichment enhances h-BN’s applicability in nuclear and thermal management contexts by leveraging its high neutron capture cross-section and slightly improved thermal conductivity. While chemical exchange distillation and laser-based methods enable enrichment, their costs limit widespread adoption to specialized applications. These considerations align with h-BN’s reported properties, such as its high in-plane thermal conductivity and low thermal expansion coefficient, reinforcing its environmental robustness and versatility.

## 6. Conclusions and Outlook

In recent years, hexagonal boron nitride (h-BN) has emerged as a highly promising semiconductor material for neutron detection applications, owing to its unique physical and chemical properties. With its outstanding thermal neutron capture cross-section, wide bandgap, high electrical resistivity, excellent radiation hardness, and superior thermal and mechanical stability, h-BN stands out as an ideal candidate for solid-state neutron detectors. These attributes position h-BN to play a key role in future applications across nuclear safety monitoring, medical imaging, aerospace radiation protection, and geophysical exploration.

Currently, high-quality h-BN crystals and thick films can be synthesized through advanced growth techniques such as high-pressure high-temperature (HPHT) synthesis, chemical vapor deposition (CVD), metal-flux methods, and physical vapor deposition (PVD). The rapid development of these methods has brought h-BN-based neutron detection research to a more mature stage. Recent studies have demonstrated that h-BN detectors utilizing enriched ^10^B isotopes can achieve thermal neutron detection efficiencies exceeding 50%, surpassing traditional ^3^He gas detectors and even outperforming semiconductor detectors based on Si and SiC in certain performance metrics. Despite this encouraging progress, the development of h-BN neutron detectors still faces several critical challenges.

Foremost among these is the issue of crystal quality and scalable production. Current synthesis techniques are still limited in terms of enlarging crystal size, reducing defect density, and improving chemical purity. Achieving the stable growth of large-area, high-quality single crystals remains a formidable challenge, particularly for methods such as flux growth and CVD, which, while capable of producing high-quality material, require further optimization to ensure batch uniformity and large-scale reproducibility. Another major bottleneck lies in the high cost associated with ^10^B isotope enrichment. Present-day enrichment techniques are complex and expensive, significantly limiting the industrial scalability of high-performance h-BN detectors. Developing more cost-effective and efficient isotope enrichment processes will be essential for reducing material costs and enabling large-scale production—an important direction for future research.

Moreover, significant improvements are still needed in detector device architecture and integration technologies. Most h-BN-based neutron detectors reported to date remain at the laboratory prototype level. To meet the stringent performance requirements for long-term stability, high sensitivity, and strong interference resistance in practical applications, further innovations in device design and fabrication processes are required. In particular, the long-term reliability and operational stability of h-BN detectors under extreme conditions—such as high temperature, high pressure, and intense radiation—must be rigorously validated before commercial deployment.

Looking ahead, research and development in h-BN-based neutron detection should focus on several key directions: (1) Advancing crystal growth techniques to enable larger crystal sizes, lower defect densities, and improved quality control. Particular emphasis should be placed on refining flux growth and optimizing CVD processes to support stable and scalable synthesis of high-quality h-BN materials. (2) Developing cost-effective, high-efficiency ^10^B isotope enrichment methods to reduce material costs and lay the foundation for industrial-scale production of h-BN detectors. (3) Optimizing device architectures and fabrication technologies, including the development of novel electrode configurations, doping strategies, and heterojunction designs to improve carrier mobility, charge collection efficiency, and energy resolution. (4) Establishing standardized fabrication and performance evaluation protocols for h-BN neutron detectors, including industry-wide benchmarks for material quality, device performance, and reliability, to accelerate their adoption in industrial, medical, scientific, and security-related applications.

In summary, while challenges remain in material synthesis, cost control, and device integration, the continued advancement of research and manufacturing technologies is poised to unlock the full potential of h-BN in neutron detection. With sustained innovation and strategic industrial development, h-BN-based detectors are expected to play a vital role in future nuclear safety, radiation monitoring, and advanced sensing systems, paving the way for next-generation, high-performance, low-cost, and reliable neutron detection technologies.

## Figures and Tables

**Table 1:** Physical properties of hBN [34,35,36].

Property		Value
Density (g/cm^3^)		2.27
Electrical resistivity (Ω/m)		10^16^
Dielectric constant (10^2^–10^5^ Hz)		4~5
Melting point (°C)		3000
Mohs hardness		1~2
Thermal conductivity	along c-axis (W/m K)	34
	in-plane (W/m K)	600
Thermal expansion coefficient (200–1000 °C)	Parallel to hot-pressing direction	7.51 × 10^−6^
	Perpendicular to hot-pressing direction	0.77 × 10^−6^
Flexural strength (MPa)	Parallel to hot-pressing direction	60~80
	Perpendicular to hot-pressing direction	40~50
Elastic modulus	Parallel to hot-pressing direction	84
	Perpendicular to hot-pressing direction	35

**Table 2:** Comparison between the carbothermal method and urea-borax method.

Method	Reaction Pathway	Limitations
Carbothermal	B_2_O_3_ + 3C + N_2_ → 2BN + 3CO (>1500 °C)	Carbon residues > 5%
Urea-Borax	Na_2_B_4_O_7_ + 2CO(NH_2_)_2_ → 4BN + Na_2_O + 4H_2_O + 2CO_2_	Requires NH_3_ atmosphere

**Table 3:** Comparison of h-BN with traditional neutron detectors.

Detector Type	Efficiency (%)	Energy Resolution (%)	Timing Resolution (ns)	Active Area
h-BN	40–96	5–10	10–100	cm^2^
^3^He	80–90	2–5	100–1000	dm^2^
Scintillators	50–80	5–10	10–100	m^2^
Silicon (B-coated)	10–30	1–2	<10	cm^2^
